# Diabetic Foot Ulcers: Current Advances in Antimicrobial Therapies and Emerging Treatments

**DOI:** 10.3390/antibiotics8040193

**Published:** 2019-10-24

**Authors:** Jesus Manuel Ramirez-Acuña, Sergio A Cardenas-Cadena, Pedro A Marquez-Salas, Idalia Garza-Veloz, Aurelio Perez-Favila, Miguel A Cid-Baez, Virginia Flores-Morales, Margarita L Martinez-Fierro

**Affiliations:** 1 Molecular Medicine Laboratory, Unidad Academica de Medicina Humana y Ciencias de la Salud, Universidad Autonoma de Zacatecas, Carretera Zacatecas-Guadalajara Km.6. Ejido la Escondida, C.P. 98160 Zacatecas, Mexico; jesusm.ra94@gmail.com (J.M.R.-A.); sergiocardenas.molecular@gmail.com (S.A.C.-C.); pe-dro_marquez@hotmail.com (P.A.M.-S.); chaure7@gmail.com (A.P.-F.); drcidbaez@hotmail.com (M.A.C.-B.); 2 Unidad Academica de Ingenieria Electrica, Universidad Autonoma de Zacatecas, Jardín Juárez 147, Centro, 98000 Zacatecas, Mexico; 3 Laboratorio de Sintesis Asimetrica y Bioenergetica (LSAyB), PA de Ingeniería Química, UACQ, Universidad Autonoma de Zacatecas, Carretera Zacatecas-Guadalajara Km.6. Ejido la Escondida, C.P. 98160 Zacatecas, Mexico

**Keywords:** diabetic foot ulcer, antimicrobial therapy, regenerative medicine

## Abstract

Diabetic foot ulcers (DFUs) are very important diabetes-related lesions that can lead to serious physical consequences like amputations of limbs and equally severe social, psychological, and economic outcomes. It is reported that up to 25% of patients with diabetes develop a DFU in their lifetime, and more than half of them become infected. Therefore, it is essential to manage infection and ulcer recovery to prevent negatives outcomes. The available information plays a significant role in keeping both physicians and patients aware of the emerging therapies against DFUs. The purpose of this review is to compile the currently available approaches in the managing and treatment of DFUs, including molecular and regenerative medicine, antimicrobial and energy-based therapies, and the use of plant extracts, antimicrobial peptides, growth factors, ozone, devices, and nano-medicine, to offer an overview of the assessment of this condition.

## 1. Introduction

Diabetic foot ulcers (DFUs) are very important microvascular diabetes-related lesions that are the consequence of several predisposed factors, such as peripheral arterial disease, bone abnormalities, diabetic neuropathy, or infections that, without appropriate management, can lead to very severe clinical conditions and, eventually, lower-limb amputation. The first cause of lower-limb amputation worldwide is diabetes, and it is reported that 15–25% of patients with diabetes present foot ulcerations over their lifetime [1]. With a certain preference for patients with type 2 diabetes, DFUs can be observed in patients with type 1 diabetes. DFUs also show an overall prevalence of 6.3%. It is a major public health issue with a very important economic impact estimated at about $8659 per patient (p/p) annually in just North America. Several reports estimated that about 85% of all patients with diabetes and lower-limb amputations were diagnosed with DFU at some point. The patients shown to be at a higher risk for DFUs are those with a low body mass index (BMI), a long clinical history of diabetes (especially old patients), and those with diabetic retinopathy or systemic arterial hypertension and a smoking history [2]. DFUs are one of the most severe complications of diabetes, and more than half of those ulcers become infected. Every single one of these infected lesions has the potential to get worse and compromise the integrity of the lower limbs. To avoid amputations and improve the patient’s quality of life, it is very important to implement a strict program for the prevention and treatment of ulcers, as well as proper management of infections [3]. Thus, it is critical to keep diabetes patients aware of new therapies and treatments and their availability in the healthcare system. The treatment of DFUs requires a multidisciplinary approach with proper medical tools, skills, and knowledge. This starts from patient education, with the application of new classifications to guide the treatment to prevent amputations. New diagnosis methods should become available, such as the 16S ribosomal DNA sequence in bacteria, to provide a better understanding of the microbiota in DFUs. It is reported that DFU has a polymicrobial nature, and, according to its geographical location, certain marked differences, wound characteristics, antibiograms according to local epidemiology, individualized antimicrobial guided therapy, regular debridement, regular assessment of wounds, and change of dressings. The latter characteristics are also aided by new biological and molecular therapies that were proven to improve infection control, the regulation of the local inflammatory profile, and improved quality of the cicatrizing process. In the next sections, this review presents an approach for the diagnosis and treatment of DFUs, focusing on the current advances in antimicrobial therapies, such as dressings, topical therapies, antibiotics, drugs, debridement methods, biological, cellular, and gene therapies, plant extracts, antimicrobial peptides, growth factors, devices, ozone, and energy-based therapies.

## 2. Diabetic Foot Ulcers

DFUs are defined as foot lesions (ulcers) that may affect the skin, soft tissue, and bone in lower limbs, causing an aggravating infection in diabetic patients that can lead to very serious consequences such as lower-limb amputations. DFUs are caused by multifactorial etiologies as part of the microvascular complications of diabetes mellitus that can lead to major amputations, in most cases by the lack of the timely and correct management of diabetic feet. Indeed, diabetes is the leading cause of non-traumatic lower-extremity amputations worldwide [1]. These serious consequences are mostly due to the absence of data on many subjects including diabetes education, preventive measures, glycemic control, comorbidities, inappropriate multidisciplinary assessment and treatment of ulcers, and later treatment failures in the prevention of ulcer recurrence [4]. Based on the 2015 prevalence data from the International Diabetes Federation, it is estimated that foot ulcers develop in 9.1 million to 26.1 million people with diabetes annually worldwide [3]. A systematic review and meta-analysis of the global prevalence of DFUs showed that the global prevalence of DFUs was 6.3%, higher in males than in females, and higher in type 2 than in type 1 diabetic patients [2]. In Mexico, there are around 12 million cases of diabetes mellitus, and since the overall prevalence of DFUs is 6%, it is estimated that more than 700,000 people are affected with any grade of DFUs [5]. DFU treatment has a high cost worldwide. In the United States (US), this cost ranges from $8000 to $17,000, depending on the grade of infection and type of amputation, with the cost rising to $43,000 in the case of partial amputation to $63,100 after major amputation [6]. All of these costs not only affect the patient’s economic and psychological status but also the family’s economy, the patient’s disability and diminished quality of life, and the finances provided by the government and health insurance intended for diabetes treatment. In patients with diabetes, it is reported that, in most cases (60–80%), the ulcers become less aggressive, and, with the proper care, they heal. On the other hand, about 10% to 15% of these ulcers remain active and 5% to 24% lead to limb amputation in approximately 6–18 months [1]. As many as 40% of patients have a recurrence within one year after ulcer healing, almost 60% have a recurrence within three years, and 65% have a recurrence within five years, making a previous incident of a foot ulcer the strongest predictor for diabetic foot ulceration [3]. The median time to healing without surgery is about 12 weeks [4]. The five-year risk of death in diabetes patients is 2.5 times higher in those with DFUs than without them, and the five-year mortality after diabetes-related amputations exceeds 70%, which is worse than in many common cancers [3,4]. Osteomyelitis is another severe complication of DFUs, and it needs to be discarded in every patient with infected DFUs. A complete assessment with the measurement of blood pressure, and laboratory testing for complete blood cell counts, creatinine, glycated hemoglobin, erythrocyte sedimentation rate, C-reactive protein level, and ankle-brachial index (ABI, normal 0.8–1.2), as well as imaging (X-ray and MRI), needs to be done, and, in some cases, a bone biopsy may also be considered. This complication reflects the poor healthcare programs in the institutions that provide primary health attention, since primary care does not commonly provide foot examinations during routine office visits, with time being a major factor impeding routine foot assessments [7].

**Figure 1:**
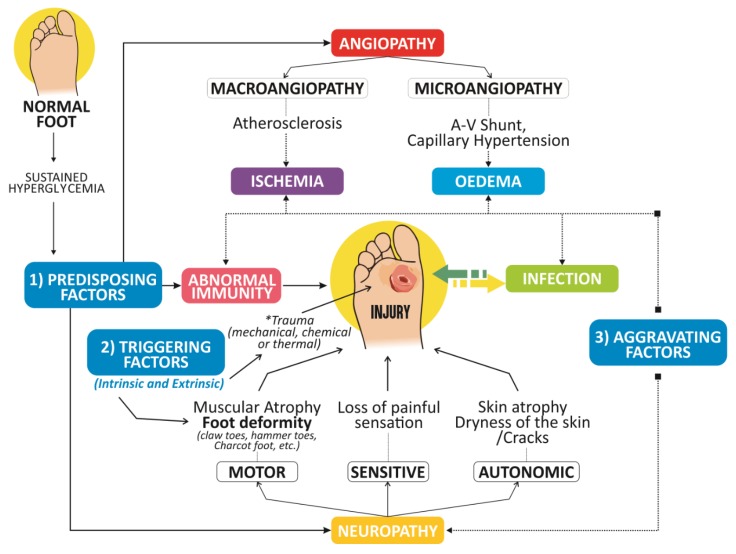
Risk factors and predisposing factors for the development of diabetic foot ulcers (DFUs). There are two main protagonists among the risk factors and/or predisposing factors of DFUs: angiopathy and neuropathy, whose presence, together with intrinsic (foot deformity) and/or extrinsic triggers, such as trauma (mechanical, chemical or thermal), causes the loss of skin integrity. Aggravating factors, such as ischemia, abnormal immunity, and neuropathy, favor the development of DFU infections.

In addition to diabetes, the risk factors for DFU include the coexistence of neuropathy (sensory, motor, and autonomous), peripheral arterial disease (PAD), immune system factors, and, in some cases, repetitive external or minor trauma (which lead to skin breakdown and ultimately to the development of infection). Bony foot deformities (such as bunions and hammertoes), which can also create points of pressure (potential ulceration sites), are also considered to be risk factors (Figure 1) [1,3,8].

It is reported that patients with severe neuropathy commonly have higher mechanical pain thresholds than diabetes patients without it [9]. The existence of infection is the most frequent cause of amputation, as infection happens in patients with severe infections, more lost tissue, and systemic organ dysfunction. Anemia (hemoglobin <11 g/dL), old age, and the presence of PAD can also participate in the progress of infection and, eventually, lead to major amputation [10]. In México, the following were reported as risk factors for major amputation: leukocytosis (>9), low serum albumin (<2), HbAc1 (>7), and chronic kidney disease in K/DOQI (Kidney Disease Outcomes Quality Initiatives), with K/DOQI-III being the most common stage. It was also found that the most affected areas by DFUs were the forefoot (48%) and the plantar region (55%) of the foot. Most patients had advanced stages of DFUs, where 93% of the lesions were grades III–V, according to the Wagner classification [11]. In 2016, a report revealed evidence that hemoglobin A1c (HbA1c) <5.8% works as a beneficial factor [12]. The factors associated with poor healing include advanced end-organ disease (congestive heart failure, peripheral artery disease, or end-stage kidney disease) [3]. Therefore, it is imperative to invest in the research for new treatments, diagnosis, and technology intended for DFUs [4]. The complexity of DFU physiopathology and the lack of uniform criteria are evident in the variety and number of existing classifications. The American Diabetes Association (ADA) recommends criteria or variables for an ideal DFU classification. These criteria include determination of the etiology, size, depth, and edema, as well as identification of perilesional damage, the state and degree of infection, vascular and neurological factors, severity of the injury, and prognosis and guidance in management and treatment. This classification should also facilitate communication between health professionals and the understanding of the patient and his family [13]. However, there are different systems of classifications for DFUs recommended by the ADA that offer guidance for the treatments needed (Table 1).

### 2.1. Physiopathology of DFUs

DFUs have a complex pathogenesis, and the main factors that influence their development are diabetic neuropathy and PAD, with trauma being a triggering factor. All of these factors together take part in different stages of ulcer development, before and after its occurrence as a delay in wound healing [3].

#### 2.1.1. Diabetic Neuropathy

Hyperglycemia produces oxidative stress (OS) on nerve cells and leads to neuropathy, which affects sensitive, motor, and autonomous nerves [14]. There is an increased production of some enzymes, such as aldose reductase and sorbitol dehydrogenase, through the polyol metabolic pathway that consumes nicotinamide adenine dinucleotide phosphate (NADPH), which is further reduced by activation of the hexosamine pathway, which limits the conversion of nicotinamide adenine dinucleotide to NADPH by inhibiting the activity of glucose-6-phosphate dehydrogenase [15]. These enzymes convert glucose into sorbitol and fructose. As these sugar products accumulate, the synthesis of nerve cell myoinositol is decreased, resulting in nerve conduction, antioxidants such as glutathione, and increased production of reactive oxygen species (ROS) [14,15,16]. Additional nerve dysfunction follows from the glycosylation of nerve cell proteins, abnormalities in the fatty-acid metabolism activation of protein kinase C, increased hexosamine pathway flux, and the polyol pathway, as well as the altered production of substance P, nerve growth factor, and calcitonin gene-related peptide, leading to further ischemia [15,16,17]. Damage to the motor neurons of the foot’s musculature may lead to an imbalance of flexors and extensors, anatomic deformities, and eventual skin ulcerations. Damage to autonomic nerves impairs sweat gland functions, and the foot may develop a decreased ability to moisturize their skin, leading to epidermal cracks and skin breakdown [16,18]. Lastly, patients may not notice foot wounds because of decreased peripheral sensation, as it depletes the foot skin of intraepidermal nerve fiber endings of the afferent A-delta and C-fibers, which are mostly nociceptors and excitable by noxious stimuli only. This affliction could be aggravated by other neuropathic conditions seen in the diabetic population, like vitamin B12 deficiency, alcohol toxicity, and end-stage renal failure [16,18]. Epidemiological studies suggest that lipid lipoproteins may contribute to PAD, as well as hypertension and smoking. Regarding motor neuropathy, the most famous feature is Charcot’s foot, which is characterized by subluxation, joint dislocation, osteolysis, bone fragmentation, and soft-tissue edema. The main issue of the foot’s structure is that muscle sheaths, tendons, and soft tissues (like plantar aponeurosis and fascia) cannot resist infections [17].

#### 2.1.2. Immunological Role in the Pathogenesis of DFUs

There are special immune features in diabetic patients that include a reduced healing response in DFUs. Some of these responses are alterations in the cellular immune response with increased T-lymphocyte apoptosis, the elevation of pro-inflammatory cytokines, and impairment of polymorphonuclear cell functions like chemotaxis, adherence, phagocytosis and intracellular killing, inhibition of fibroblast proliferation, and impairment of the basal layer of keratinocytes with reduced epidermal cell migration, which inhibits wound healing [14,16]. High blood glucose is also a good medium for the growth of bacteria, mainly aerobic Gram-positive cocci like *Staphylococcus aureus* (*S. aureus*) and β-hemolytic streptococci [14,16]. The metabolic dysfunction seen in diabetes impairs the synthesis of proteins, fibroblasts, and collagen, as well as further systemic deficiencies. Impairment of the immune system with serum glucose levels of ≥150 mL/dL was also described [14,16]. The common consequence of these features is a prolonged inflammatory state.

#### 2.1.3. PAD

It is documented that 78% of patients with DFU also have PAD [19]. Hyperglycemia induces changes in the foot’s peripheral arteries and begins at a cellular level. Endothelial cell dysfunction is the most important feature of microcirculation dysfunction, as it leads to a decrease in vasodilators, particularly in the synthesis of nitric oxide. Plasma thromboxane A2 levels become elevated with consequent persistent vasoconstriction and plasma hypercoagulation, leading to an increased risk of ischemia and ulceration [14,16]. In the endothelium, there are changes in the proliferation of endothelial cells, thickening of the basement membrane, increased blood viscosity, alterations in microvascular tone, smooth muscle cell proliferation, decreased antioxidant capacity, and decreased local angiogenesis [14,16].

### 2.2. DFU Infection

According to The International Working Group on the Diabetic Foot, infection is the invasion and multiplication of pathogenic microorganisms within tissues of the body [20]. Diabetic foot infections (DFIs) increase morbidity and can lead to limb amputation. Infections in DFUs are frequent and serious complications of ulcers [21,22]. It is estimated that 50% of DFUs are infected upon presentation [21,22], and 80% of non-traumatic lower-limb amputations are a consequence of DFU infection [21,22]. Patients with DFIs are usually hospitalized multiple times and are often exposed to multiple courses of antibiotics [23]. Wound infections are a factor in the delay in the healing process, and, if they are not treated properly, they could lead to systemic compromises [21,22]. Various aspects of wound microbiology are responsible for the development of foot infection. These include microbial load, the diversity of microbes, the existence of infective organisms, and the synergistic association amongst microbial species. Infection is said to occur when the microbial load is greater than 105 organisms per gram of tissue [17]. The exposed tissue left by DFUs then becomes a target for skin commensal bacteria that can colonize the wound, even though, since colonizing does not have a proper host immunological response, it cannot be called an infection [24]. Figure 1 shows that the triggering factor is external. These factors can be physical, chemical, and mechanical. Ischemia, neuropathy, edema, infection, and a poor immune response trigger a complex and very difficult to heal wound or ulcer [25] that is predisposed to infections in the diabetic foot. It is critical to assess ulcer infection based on the advice of the Infectious Diseases Society of America (IDSA) and the classification of DFI [16]. The diagnosis of infection is performed by clinical observation and is based on the presence of at least two of the following signs: inflammation, induration, erythema perilesional, hyperesthesia, pain, local heat, and purulent exudate (Table 1) [26]. It is documented that 78% of patients with DFU also have PAD [19]. Endothelial cell dysfunction is the most important feature of microcirculation dysfunction, as it leads to a decrease in vasodilators, particularly in the synthesis of nitric oxide. Furthermore, plasma thromboxane A2 levels become elevated with consequent persistent vasoconstriction and plasma hypercoagulation, leading to an increased risk of ischemia and ulceration [14,16].

#### 2.2.1. Microbiota in DFUs

The microbiota in DFUs was largely studied. This microbiota mostly relies on the host’s immune status and their physiopathological features. The predominantly identified bacteria in DFUs are shown in Table 2.

The polymicrobial nature (constituting Gram-negative and Gram-positive bacteria, anaerobic bacteria, and certain fungi) of chronic wounds like DFUs, recently described using molecular methods, is a barrier against the traditional bacterial culture methods that, for a long time, targeted what was believed to be the only microorganism present (Gram-positive bacteria) [24]. A microbial population difference was also reported between diabetic and non-diabetic ulcers, and the bacteria found were Gram-negative and Gram-positive [33]. Another author reported the predominance of Gram-negative over Gram-positive bacteria (59% and 41%) in a microbiological evaluation of DFI [27]. There are also “favorite spots” for microorganisms in the DFUs, which are marked by their oxygen consumption. For example, aerobic bacteria are localized in the upper surface where oxygen content is relatively high, while anaerobes are localized more deeply in the niches created by aerobic oxygen consumption [34]. Of all the microorganisms present in DFUs, the most commonly isolated Gram-positive bacterium worldwide is *Staphylococcus aureus* and the most commonly isolated Gram-negative bacterium is the *Pseudomonas* species (spp.), followed by *Escherichia coli*, *Proteus* spp., *Enterobacter* spp., and *Citrobacter* spp. [23,27,28,29]. In a microbiome characterization study of new and recurrent DFUs using 16S amplicon sequencing (16S AS), *S. aureus* was isolated in 72% of culture-positive samples, whereas the most commonly detected bacteria in all ulcers were *Peptoniphilus* spp., *Anaerococcus* spp., and *Corynebacterium* spp. [35]. Geography plays an important role in the etiology of DFUs. It is reported that in Western countries, Gram-positive aerobic cocci are the main microorganisms; however, in warmer places (particularly in Asia and Africa), Gram-negative bacilli are more prevalent. In Mexico, the main bacterium isolated by standard methods was *S. aureus,* [34]. In Bangladesh, it was found that the most common bacteria in DFUs samples were *Pseudomonas* spp. (22/29%), *Enterobacter* spp. (22/7%), and *Staphylococcus* spp. (13/13%) [32]. Furthermore, in a study performed in India, Gram-negative pathogens were reported to be the most predominant (58.5%) [31], thereby demonstrating the prevalence of Gram-negative bacteria in Eastern countries. The main anaerobes isolated from up to 95% of deep diabetic wounds are *Peptostreptococcus* spp., *Bacteroides* spp., and *Prevotella* spp. [24], predominantly seen in DFIs with ulcers that are deeper, more chronic, and associated with ischemia, necrosis, and gangrene or foul odor [30,31].

#### 2.2.2. Biofilm

By definition, biofilm is a highly organized arrangement of bacterial communities that are rooted in an extra polysaccharide matrix with transformed phenotype and growth patterns. The formation of biofilms is another factor that leads to the chronicity of diabetic foot wounds. Biofilm makes the wound healing process very slow and infection very difficult to resolve, as local access for antimicrobial agents and the host’s immune system is hampered. A prospective study reported that biofilms were formed predominantly by *Staphylococcus aureus*, and the organisms causing chronic DFUs were commonly multidrug-resistant [17,24,28].

#### 2.2.3. Diagnosis of DFU Infections

There are two principal approaches to the diagnosis of DFU infections: microbiological and molecular approaches.

##### Microbiological Approaches

It is crucial to isolate the causative microorganisms of DFIs to engage in appropriate treatment. Four major techniques are used to collect samples from deep tissue wounds. These techniques include needle aspirates, swabs, a tissue biopsy (the most advantageous and standard method), and curettage after debridement. Due to the fear of infectious growth and the loss of adjacent ischemic or healthy tissue, biopsies are a very difficult and delicate procedure. On the other hand, swab cultures are manageable since sample collection becomes easier and can be taken from any kind of wound. However, swab cultures are sometimes not reliable since they generally include the colonizing but not the causative pathogens. Traditional wound swab cultures do not correlate well with tissue biopsy cultures and often lead to overuse and non-directed antimicrobial therapy, incrementing bacterial multi-resistance. Therefore, sample collection techniques play a crucial role in bacterial culture identification [17,24]

##### Molecular Approaches

Molecular biology tools provide a powerful means to define microbial communities in chronic wounds. Significant microbial diversity can be revealed in a single clinical sample by using culture-free sequencing of bacterial DNA. The identification of the bacterial microbiome was made possible by the discovery of the 16S ribosomal DNA sequence, known as “the universal primer”. The identification of bacterial DNA is carried out by the amplification and sequencing of 16S DNA. Then, a comparison is made between the identified flanking sequences and a group of known bacterial sequences from a virtual library, which then determine the bacterial species; with some standards, it is possible to estimate the bacterial load. One of the most important advantages that molecular approaches have over traditional bacterial cultures is the time spent in bacterial identification, because the detection of microbes is possible on the same day the sample is collected, without the time required for bacterial growth in a culture or the environmental selection pressures inherent to the culturing processes. Molecular methods are progressing and becoming more accessible and affordable. It is now possible to use bacterial DNA from a wound site to identify the pathogens present; Hence, this method should be available for most of the diabetic community, to enable a better microbial assessment of wounds [17,24].

#### 2.2.4. Multidrug-Resistant Bacteria

Bacterial multidrug resistance to potent and new drugs challenges clinical criteria and the systematization of the knowledge and care of patients. Multidrug-resistant bacteria are being developed around the world, mostly due to the inappropriate use of antibiotics [23]. Empirical antimicrobial therapy is a riddle based on experience and should cover resistant strains of germs, such as the methicillin-resistant *Staphylococcus aureus* (MRSA), *Pseudomonas*, and anaerobes. Openly published evidence suggests that there is no reason to prescribe antibiotic therapy for an uninfected foot wound as either a prophylaxis against infection or in the belief that it accelerates the healing of the wound [36]. In a study performed in Bangladesh, most of the isolates from DFI patients were commonly resistant to cephalosporin (ceftazidime, ceftriaxone, cefuroxime) and carbapenem (aztreonam) [32]. In Mexico, the least effective antibiotics for Gram-positive bacteria identified in DFIs were penicillin and dicloxacillin; for Gram-negative bacteria, cefalotin and penicillin were the least effective. Levofloxacin and cefalotin, and amikacin were the most effective antibiotics for Gram-positive and Gram-negative bacteria, respectively, and 50% of the strains were allegedly resistant to vancomycin [34]. Table 3 displays a summary of the most common bacteria isolated from DFI and the more or less efficient antibiotics in each geographic localization.

## 3. DFU Infection Management Therapeutic Approaches

DFIs can lead to partial or total foot or limb amputation or, in severe cases, the death of the patient. Paired with ischemia, an infected diabetic foot remains one of the biggest challenges in the management of DFUs [37].

The accumulation of the bacterial load in the wound leads to the production of local and systemic cytokines that can produce a systemic inflammatory response (SIR) and shock, thereby highlighting the importance of infection management for DFUs [38]. Several antimicrobial therapies and physical interventions are routinely used depending on the severity of infection, from topical and oral treatments, aimed at mild to moderate infections, to intravenous therapies, which target more severe infections. Once started, an antibiotic course needs to be continued until all clinical signs resolve and laboratory tests fall within the non-pathological range [7]. During the treatment of infection, the wound should be regularly monitored (during each dressing change or on a bi-weekly schedule) to evaluate the effectiveness of the therapy [39]. A summary of the antibiotic regimen used in DFIs is shown in Table S1 (Supplementary Materials).

### 3.1. Debridement

Debridement is the removal of the bacterial biofilm and necrotic tissue from a wound and is one of the key components of foot ulcer infection management [40]. It facilitates the complete assessment of the wound, provides tissue for microbiological culture, and promotes wound healing [1]. The accumulation of necrotic tissue around the wound area is a part of the normal healing process. However, excess necrotic tissue hinders the formation of new tissue, which is why debridement aids in increasing the speed of wound healing [41]. Debridement and cleansing of the wound are necessary companions to antibiotic treatment and are usually performed using isotonic saline solutions (0.9% NaCl) [7]. In addition, sharp debridement decreases the bio-burden of the hyperkeratotic margins typical of plantar neurotrophic ulcers. This process should be performed every seven to 14 days [1,8]. In the clinic, two types of debridement techniques are used: active and autolytic [39]. Active debridement involves the physical removal of necrotic material by manual techniques, for example, surgical debridement, which uses a scalpel and tweezers to remove dead tissue, usually causing bleeding of the wound bed. Hydro-surgical debridement removes dead tissue by using a strong jet of water [39]. Ultrasonic-assisted debridement is convenient for its use in outpatient settings. This process consists applying low-frequency waves (25 kHz) with irrigation fluids [39]. Autolytic debridement is performed by enhancing the moisture of the wound area to promote the natural shedding of tissue, which is usually achieved by applying hydrocolloids and hydrogels. A recent study compared clostridial collagenase ointment (CCO) for enzymatic debridement to standard care plus hydrogel and found no difference in the wound size at six and 12 weeks [8].

### 3.2. Dressings

The role of the wound dressing is to protect the area from infection and environmental exposure, as well as promote the area’s moisture to facilitate new tissue formation and autolytic debridement. As previously mentioned, autolytic debridement enhances the breakdown of necrotic tissue through endogenous proteolytic enzymes [8]. Some of the existing dressing types include films, hydrogels, acrylics, hydrocolloids, calcium alginates, hydrofibers, and foams. Wounds with high secretion levels need absorbent dressings, whereas a dry wound requires moisture balance dressings that provide moisture to the wounds [7]. All types of dressings show similar rates of healing promotion [8]. Some of the most widely used types of dressings in clinical environments are explored below.

#### 3.2.1. Hydrogels

The composition material of hydrogel dressings includes insoluble copolymers capable of binding water molecules. The water in the matrix can be donated to wounds and, conversely, the matrix is capable of absorbing wound exudates, thereby maintaining an optimal level of moisture in the wound [8]. Some evidence suggests that hydrogel dressings are more effective in healing DFUs than other dressings [8].

#### 3.2.2. Alginate Dressings

Alginate products (calcium alginate, calcium sodium alginate, or alginic acid) are derived from seaweed. These products act similarly to hydrogels by absorbing wound exudates and maintaining a moist wound environment [8]. Previous reviews and meta-analyses showed no significant difference with basic contact dressings or silver hydrocolloid dressings [8].

#### 3.2.3. Acrylics

The dressing (usually a thin clear film) is permeable to water vapor. However, it has a low absorbance capacity and can pose removal difficulties [7].

#### 3.2.4. Hydrocolloids

The composition of this type of dressing involves hydrophilic carboxy components and hydrophobic methylcellulose bound to a polyurethane film. These components promote autolytic debridement; they are also self-adherent and long-wearing. However, this process can disturb the wound area during removal, and allergic reactions can develop [7].

#### 3.2.5. Foam Adhesive

This type of adhesive is composed of absorbent polyurethane with different pore sizes and can serve as a vehicle for silver and ibuprofen onto the wound. However, foam adhesives have the disadvantage of inducing macerations in the surrounding skin [7].

#### 3.2.6. Hydrofibers

Hydrofibers are composed of carboxymethylcellulose sheets. Some advantages are their highly absorptive capacity and ease of removal. However, a secondary dressing is needed [7].

### 3.3. Topical Antimicrobials

Topical antimicrobials are not a preferred treatment for chronic wounds due to their lack of contribution to moisture balance maintenance and autolytic debridement, as well as the potential for the development of contact dermatitis. When used, topical antimicrobials are selected based on their low toxicity to the host tissue. In the paragraphs below, some topical antiseptics/antimicrobials available for DFIs are described.

#### 3.3.1. Povidone Iodine 10% Solution

Povidone iodine is a broad-spectrum antibacterial agent that can penetrate the bacterial biofilm and promote wound healing. It is typically used as a short-term treatment and reassessed every two to four weeks. However, chronic use can cause thyroid dysfunction, and it can be toxic to granulation tissue.

#### 3.3.2. Chlorhexidine

This agent has a broad antibacterial effect and promotes wound healing. However, it may damage cartilage tissue [7].

#### 3.3.3. Acetic Acid 5%

This is a useful treatment against bacteria from the genus *Pseudomonas* and other Gram-negative bacteria. It can produce tissue toxicity and cause fibroblast growth inhibition [7].

#### 3.3.4. Silver Compounds

Foams, calcium alginates, hydrofibers, hydrogels, sheets, silver sulfadiazine cream, and silver nitrate sticks produce activities against *Escherichia coli*, *Klebsiella*, *S. aureus*, and methicillin-resistant *Staphylococcus aureus* (MRSA), and also have antifungal and antiviral properties. These compounds may have toxicity to the re-epithelialization process, leading to delayed healing.

#### 3.3.5. Sodium Hypochlorite (Bleach)

Bleach has a broad antibacterial effect, but it is an irritant with high tissue toxicity, which inhibits fibroblasts, and is best used as a disinfectant and not for wound care.

#### 3.3.6. Benzalkonium Chloride

Benzalkonium chloride has a broad antibacterial effect and antifungal effect and has the same adverse effects as sodium hypochlorite.

#### 3.3.7. Hydrogen Peroxide

This type of peroxide has activities against Gram-positive bacteria. Its main adverse effect is a risk of bullae formation [7].

Other topical antimicrobials that were studied but not found to have clear benefits include cadexomeriodine, carboxymethylcellulose hydrofiber, superoxidized solutions, tobramycin beads, and chloramine treatment [8].

### 3.4. Systemic Antibiotic Therapy

Systemic antibiotic therapy is indicated when signs of localized, advancing, or systemic infections are present. The route of administration and type of antimicrobial agent to be used are determined by the results of a microbiological culture, the severity of the clinical signs, the body structures involved, and the immunocompetence of the patient [39]. During routine care, broad-spectrum antibiotics are typically used first, before switching to a more targeted agent once the bacterial culture results are available. In severe, non-responsive, or spreading infections, or where serious osteomyelitis is suspected, hospitalization and intravenous (IV) antibiotic therapy may be done [39]. Oral antibiotic therapy covers activities against Gram-positive staphylococci and streptococci. If a single agent fails to address the infection, a second antibiotic is added. Empiric therapy against methicillin-resistant *Staphylococcus aureus* (MRSA) is considered if the patient has a previous history of infection, if there is a high incidence of MRSA infection in the population, or if the infection is resistant to treatment [39]. IDSA recommends one to two weeks of antibiotics for mild infections and two to three weeks for moderate-to-severe infections, but antibiotics can usually be discontinued once the clinical signs and symptoms of infections resolve [8]. The most commonly used broad-spectrum agents are carbapenems β-lactam, or β-lactamase inhibitor combinations, such as piperacillin/tazobactam, ampicillin/sulbactam, and ticarcillin/clavulanic acid.

Carbapenems are a mainstay in the treatment of multidrug-resistant Gram-negative bacteria; however, resistance to this group of drugs is increasingly being reported in the clinic [32]. Anaerobic bacteria are preferentially treated with metronidazole, which is also used for the management of chronic DFU infection [31]. Current guidelines suggest cefoperazone/sulbactam or piperacillin/tazobactam with clindamycin as the empiric antibiotics of choice for DFIs, with an escalation to carbapenem (meropenem) with teicoplanin depending upon the culture’s sensitivity report [32]. In the SIDESTEP study (ertapenem versus piperacillin/tazobactam for diabetic foot infections), the authors compared the clinical success rates between ertapenem and piperacillin/tazobactam, and the results were similar (94.2% vs. 92.2%). Although ertapenem does not provide coverage for *Pseudomonas* or enterococci, the clinical response for the patients from whom these organisms were isolated was similar [42]. Additional agents and combinations used in the clinic include cefepime plus tazobactam, imipenem, amikacin, and gentamicin [16]. Antimicrobial therapy, along with surgical treatment or debridement, is essential for treating any chronic deep infections in the bone [43]. MRSA is a serious problem in hospital settings that regularly affects patients with DFUs. The most widely used agent in MRSA treatment is vancomycin, although a 50% increase in reports of resistance to this drug led to the use of linezolid as an alternative therapy. Linezolid is an oxazolidinone and has activity against Gram-positive organisms, such as staphylococci (including both MSSA and MRSA isolates), streptococci, and enterococci, including vancomycin-resistant isolates (VRE). Although linezolid is not acknowledged by the US Food and Drug Administration for use against osteomyelitis, it does penetrate bone [43].

DFIs can present a complication known as skin and skin structure infections (cSSSIs). The treatment of choice for this diagnostic is a piperacillin/tazobactam combination and linezolid. Specifically, for osteomyelitis associated with overlying DFI, there are currently no approved drugs [44].

### 3.5. DFU Emerging Therapies

#### 3.5.1. Drugs

There are various emerging therapies that are different from the standard care for DFUs, whose main objective is to accelerate ulcer healing. Some examples of these treatments are adjuvant growth factors, inflammatory modulators, plant extracts, blood products, biologic therapy, wound negative pressure, hyperbaric oxygen therapy, and skin substitutes. However, these therapies are companion therapies and do not replace standard care for diabetic foot problems. Some emerging therapies are explored below in more detail.

##### Ciprofloxacin-Loaded Calcium Alginate Wafer

A ciprofloxacin-loaded calcium alginate wafer was prepared in a previous study. This study evaluated the application of this wafer directly to the wound site. The dressings showed an initially fast release followed by sustained drug release, which could inhibit and prevent re-infection caused by both Gram-positive and Gram-negative bacteria. The dressings also showed biocompatibility (>85% cell viability over 72 h) with human adult keratinocytes [45].

##### WF10 (Immunokine, Nuvo GmbH)

WF10 is an aqueous solution (1:10) of the chlorite drug OXO-K99, which contains 4.25% chlorite, 1.9% chloride, 1.5% chlorate, and 0.7% sulfate with a sodium cation. The chlorite ion is the active principle and is used clinically for chronic inflammatory disorders, such as proctitis and cystitis. WF10 has anti-inflammatory and antiseptic properties, which are mediated by an increased immune response, as WF10 stimulates the phagocytic activity of macrophages via the myeloperoxidase–hydrogen peroxide–halide system. In a study by Yingsakmongkol, WF10 was administered alongside standard treatment for severe DFUs. This study documented that neuropathic ulcers achieved either a good or fair outcome, with 81% achieving a good outcome [46].

##### Pirfenidone (PFD)

PFD is a modulator of the extracellular matrix and is an antifibrogenic molecule used for the treatment of idiopathic pulmonary fibrosis. PFD has antioxidant and anti-inflammatory properties and reduces secreted and cell-associated tumor necrosis factor-alpha (*TNF-α*) levels. In a randomized double-blind trial conducted in Mexico, the efficacy of topical PFD + M-DDO (an antimicrobial and antiseptic agent) versus ketanserin, a quinazoline derivative, a serotonin antagonist of *5-HTR2*, with no agonistic properties (approved for wound treatment by the Mexican Comisión Federal para la Protección contra Riesgos Sanitarios: COFEPRIS), was evaluated in the treatment of non-infected chronic DFUs. Patients received PFD + M-DDO or ketanserin for six months. Patients receiving PFD treatment had improved levels of *TGF-β1*, which is normally decreased in diabetes, and which promotes the differentiation of fibroblasts to myofibroblasts and cell proliferation and stimulates keratinocytes to produce laminin and other constituents of the normal basement membrane [47].

##### Deferoxamine (DFO)

DFO is an iron chelator that was used as a hypoxic-mimetic agent. DFO induces hypoxia-inducible factor 1-alpha (*HIF-1α*) accumulation under normoxia [48]. *HIF-1α* mediates various processes, including cell metabolism, proliferation, survival, and angiogenesis, and regulates a number of target genes, such as vascular endothelial growth factor (*VEGF*), erythropoietin (*EPO*), and stromal cell-derived factor-1a (*SDF-1a*). In a previous study, DFO significantly increased neovascularization through the upregulation of *HIF-1α* and target genes, including *VEGF* and stromal cell-derived factor-1α (*SDF-1α*). Two different studies showed that the administration of DFO to diabetic wounds improved wound healing, along with enhanced granulation tissue formation, re-epithelization, and neovascularization [48,49].

##### Nitroglycerine (Isosorbide Dinitrate)

Nitroglycerine can be employed as an effective donor of nitric oxide (NO) to diabetic wounds, leading to increased blood flow and biochemical activity of the ulcers and facilitating wound healing [50].

#### 3.5.2. Biologics

Biologics are drugs isolated from natural sources, including humans, animals, and microorganisms (vaccines, blood and blood components, and gene therapy). Within wound care, biologics include products like cell-based and growth factor therapies. Biologics are regulated by the FDA’s Center for Biologics Evaluation and Research [51].

##### Growth Factors and Proteins

Table 4 summarizes the different biologics used as therapies for the treatment of DFUs, which comprise several proteins and growth factors.

##### Growth Factors

Growth factors, such as the growth factor derived from platelets-BB (PDGF-BB), fibroblast growth factor β (FGFb), epidermal growth factor (EGF), VEGF, and granulocyte colony-stimulating factor (G-CSF), among others, are used to accelerate the healing of wounds. There is currently insufficient data regarding their efficacy, and they are not widely available. Some of these factors are combined with different extracts and molecules to yield a synergy of activity [52]. PDGF-BB is the most widely studied growth factor in wound healing and is currently approved for clinical use in recombinant DNA technology [53]. As PDGF treatment of diabetic wounds showed promising results, other growth factors started to be tested in the clinic [54]. Kusmanto et al. conducted a double-blind study where the efficacy of VEGF was compared against a placebo. The study reported a more significant DFU reduction (60%) in patients treated with VEGF than in those treated with placebo [52]. After VEGF, FGFb shows the most potent mitogenic activity. FGFb showed its usefulness in the treatment of DFUs. The intra-lesion administration of FGFb over an eight-week treatment course showed an improved reduction of ulcer size [55]. EGF was also used in several clinical studies in advanced DFUs, and its administration showed promising results in the formation of granulation tissue and the prevention of amputation in patients [56]. Intra-lesion injection of the recombinant form of EGF (rhEGF) directly at the site of the wound demonstrated a greater pharmacodynamic response in terms of granulation tissue growth and wound closure [44]; while PDGF-BB, FGFb, VEGF, and EGF were the most frequently used factors to improve the wound’s healing time, there were also clinical trials studying growth factors in the context of infection management. For instance, G-CSF was used in patients with ulcers complicated by soft-tissue infection, but it did not show a benefit in the treatment of the infection or cure the ulcer. However, other studies suggested that the use of therapy with G-CSF may have a benefit in reducing major amputations [57].

##### Alpha Connexin Carboxy-Terminal (ACT1)

ACT1 is a peptide mimetic of the C-terminus of *Cx43*. ACT1 has roles in dermal wound healing and re-epithelialization. Its use is correlated with increases in the transforming beta growth factor (TGF-β) messenger RNA (mRNA) and collagen a-1, and decreases in chemokine ligand-2 and recombinant human TNFα, resulting in the promotion of angiogenesis, fibroblast migration, and keratinocyte proliferation, and a decrease in infiltrating neutrophils and macrophages at the wound site. In a prospective randomized control trial, ACT1 was evaluated using a clear topical gel formulation (1.25% hydroxyethyl cellulose) containing ACT1 (100 mmol/L), which was applied topically. The acceleration of the healing of chronic DFUs when incorporated into standard of care protocols was measured. ACT1 treatment was associated with a higher reduction in the mean ulcer area with a mean baseline of 12 weeks (72.1% vs. 57.1%). None of the reported adverse effects were treatment-related, and ACT1 was not immunogenic [58].

##### Insulin

Insulin is the universal treatment for diabetes since the 20th century, since it is a physiological glucose-lowering agent. The use of topical insulin recently became of greater interest as a healing agent in DFUs [64]. Different presentations of insulin, such as insulin-based sprays, creams, and dressings, showed great success in treating the chronic ulcers of patients with diabetes mellitus, as well as in animal studies [59]. Unfortunately, the use of topical insulin presents a great challenge due to the instability of the molecule.

##### Neuropeptides

Peripheral nerves and cutaneous neurobiology contribute to the normal healing of wounds by maintaining a bidirectional connection between the nervous system and the immune system. It is known that diabetic peripheral neuropathy (DPN) affects these signaling pathways, thereby contributing to chronic wounds and ulcers. Studies showed that the impaired secretion of neuropeptides by C-nociceptive fibers (which are secondary to neuropathy) negatively affects the progress of healing [59]. Neuropeptides like calcitonin gene-related peptide (CGRP), corticotropin-releasing hormone (CRH), melanocyte-stimulating hormone (MSH), pituitary adenylate cyclase-activating polypeptide (PACAP), proopiomelanocortin peptides (POMC peptides), secretoneurin (SN), urocortin, vasointestinal polypeptide (VIP), and neurotensin (NT) serve as markers in the diagnosis of infection in the most severe stages of foot diabetic disease, especially in osteomyelitis. They are also involved in the activation of growth factors that help wound healing [65].

##### Antimicrobial Peptides

Currently, the emergence and spread of bacteria resistant to conventional antibiotics constitutes a rising global threat. For this reason, the development of alternative compounds is urgently required [66]. Antimicrobial peptides (AMPs) are effector molecules of the innate and adaptive immune system that are found in almost all organisms [67]. AMPs are short polypeptides (generally no greater than 60 amino acids) that share a cationic character and an amphipathic structure [67,68]. They have variable mechanisms of action, either acting at the membrane level or internally, which affect the synthesis of proteins and the DNA replication of the pathogen. In this way, they help the host to regulate various mechanisms including the processes of inflammation and wound closure (Table 5 and Table 6) [67,69].

The AMP spectrum of activity is broad, mainly comprising antiviral, antifungal, antibacterial, and antitumor activity [70]. Their use as a monotherapy for infection management, their combination with conventional antibiotics for synergistic purposes, their application as immunomodulators, and their use as neutralizing endotoxins continue to be explored [69]. Currently, most AMPs are used in some phase of clinical studies. For example, the FDA classified Neuprex (RBPI 21) as an orphan drug [71]. Neuprex’s level of toxicity is unknown, and studies only evaluated a topical route for its administration [67]. Additionally, the spermatozoa embryotoxic and paralyzing activity of peptides, such as nisin and magainin, whose effects can work as a vaginal contraceptive method, was reported [68,70]. Mammalian AMPs can be observed in cells like neutrophils, specifically in their granules, which contain defensins, lysozymes, indolizidine, lactoferrin, cathelicidins, and bactenecins. They can also be detected in epithelial cells [72].

To date, these peptides were classified as natural peptides, which are obtained synthetically or from microorganisms. In recent cases, the antimicrobial peptides of mammals were of interest, especially the α-defensins (classical) found in neutrophils and Paneth cells and the β-defensins found in leukocytes, neutrophils, and skin cells [72]. Some AMPs were assessed in the closure of diabetic wounds of mice, thus confirming their effectiveness. Human LL-37 cathelicidin is among the most powerful endogenous peptides, since a study in 2008 reported that transferring the adenovirus-mediated LL-37 to excisional wounds in diabetic C57BL/6J-ob/ob mice improved their re-epithelialization and granulated tissue development [73]. Later, it was confirmed that the recombinant peptide PLL-37 (derived from LL-37 with the N-terminal proline) increased re-epithelialization and angiogenesis in wounds with diminished healing [74].

In 2012, LL-37 with IDR-1018 (innate defense regulatory peptide) was analyzed, confirming that IDR-1018 was less toxic than LL-37 in vitro, and the healing of non-diabetic murine and porcine infected wounds with *S. aureus* was significantly stimulated [75]. Treatment with LL-37 is secure and well tolerated in patients with venous leg ulcers, where its effect on wound healing was observed [76]. A different study uncovered an AG-30 helical antimicrobial peptide with angiogenic properties, which can also be attributed to angiogenic and antibacterial properties. This was observed in an in vivo model of the wound healing of diabetic mice with a methicillin-resistant *S. aureus* infection (MRSA) [76].

Pexiganan (MSI-78) is a different antimicrobial peptide whose therapeutic potential on DFUs is internationally accepted; it is isolated from the skin of the African clawed frog, *Xenopus laevis* [77]. The application of 1% pexiganan acetate cream was advertised for the topical treatment of mild-to-moderate DFUs. However, its effectiveness was not superior to that of the classic oral antibiotics [78]. Another antimicrobial peptide is SR-0379, which was investigated in a multi-center, double-blind clinical trial on 12 patients with DFU and was confirmed to be effective in closing diabetic wounds; it was also proven to be well tolerated and safe [79]. A different study published guar gum as an administration system for nisin, which is an antimicrobial peptide, and revealed that nisin maintained its antimicrobial activity when it was included in guar gum gel, and it was also found to act on established biofilms [80].

##### Platelet-Rich Plasma (PRP)

In the current decade, many reports suggested the administration of platelets or the supernatant (obtained from the platelet suspension) to improve wound healing. This suspension of blood plasma enriched with a high concentration of platelets and abundant platelet growth factors also has a fraction of autologous blood platelets, which contain various growth factors and cytokines. PRP stimulates wound healing by bringing undifferentiated cells to the site of the lesion after triggering cell division. It is durable and profitable compared to recombinant human growth factors; in addition to being an autologous source of factors, it is also free of transmissible pathogens [81]. The signaling of the platelet proteins in the PRP attracts macrophages and plays an important role in the host’s defense mechanism at the wound site. The antimicrobial properties of PRP were confirmed in an evaluation against *E. coli*, MRSA, *Candida albicans*, and *Cryptococcus neoformans* [82,83]. The use of different kits for obtaining PRP was reported. However, it was not mentioned for what type of treatment each extraction kit was used. Therefore, is important to know the types of kits used to obtain the platelets and their use in different treatments [84]. In the specific case of the treatment of chronic cutaneous ulcers with PRP, PRP can work well even when the ulcers are from different etiologies. This study revealed an ulcer reduction of 0.48 cm per week and an average healing time of 6.11 weeks [85], inferring that PRP therapy for chronic ulcers is an efficient and reliable treatment. A study on the treatment of neuropathic DFUs was conducted in Iran, using the PRP Secollow Kit SK50-20. This study observed a decrease in the ulcers in all groups after seven weeks, with total healing observed after eight weeks, and, upon monitoring the patients afterward, no recurrence was observed [86]. Another report mentioned a clinical case of a 71-year-old type II diabetes patient who suffered severe injuries in his feet due to his inability to feel high temperatures. The patient was hospitalized immediately, and no treatment seemed to help him. Consequently, treatment with PRP was chosen. The extraction of PRP was made with a standard PRP protocol (Bio-Acting BIoJel Inc.), and the isolated PRP was implanted 4 mm into the wound. Following 20 days, the tissue started to exhibit regeneration and was totally restored at eight weeks [87]. Different authors studied the treatment of pilonidal sinus disease in healing wounds using PRP gel as a therapy. They revealed that the gel provoked faster healing (within 12 weeks). Therapy with PRP gel for pilonidal sinuses is designated as a different form of efficient and fast healing for any scar [88]. Other research observed a significant improvement in the healing process in the group that was treated with the PRP gel, with 86% vs. 68% from the control group. The PRP gel was more efficient in healing DFUs, exhibiting a higher healing rate and limiting infections in the ulcers [89]. Both PRP therapies, injectable and gel, demonstrated to be a novel alternative for managing chronic wounds, highlighting the faster rate of healing and their antimicrobial activity. However, further studies are required to establish the real effectiveness of PRP against wounds.

##### Cell and Gene Therapy

Cell and gene therapy are in-development techniques used to improve DFU treatments. Stem cells, keratinocytes, and fibroblasts were investigated as treatments for chronic wounds. Stem-cell therapy is employed to increase blood flow in limbs with ischemia. It is thought that this procedure can help to heal chronic wounds, but studies of this treatment are currently more theoretical [90,91]. Encouraging results were observed in several studies using autologous stem cells, mesenchymal bone marrow cells, and mononuclear cells derived from bone marrow for DFU healing [92].

1. Stem Cells

Two of the singular features of stem cells are their self-renewal and the differentiation into several cell lines. Among the different types of stem cell therapies are mononuclear cells derived from bone marrow (BM) and mesenchymal stem cells (MSC). MSCs contain multipotent progenitors and can differentiate into several cell lines [93]. In a recent study, MSCs in a collagen matrix were used to assess the progression of DFU healing in a murine model. The conclusions were satisfactory, with greater healing found in mice treated with MSCs compared to the control mice [94].

2. Fibroblast Cultures

The application of dermal fibroblasts, secretory collagen and matrix proteins, growth factors, and cytokines capable of generating a three-dimensional dermis substituted as a graft was employed to manage non-ischemic ulcers [95]. An analysis of fibroblasts/keratinocytes (Apligraf^®^, Graftskin^®^) reported satisfactory results. Unfortunately, studies for the management of chronic wounds are very limited in the application of this kind of therapy; thus, it is necessary to conduct further research to strengthen and clarify the scenarios for these novel therapies [96].

3. Grafting (Bioengineering)

In DFUs, grafting can be used to reconstruct skin defects at higher activation rates. The application of grafting is restricted to external injuries that barely affect the skin and not the soft tissues, muscles, joints, or bones [96].

4. Bovine Fluid Collagen

Bovine fluid collagen is a well-refined fibrillar bovine collagen fluid. Unlike traditional collagen in biological scaffolds (cross-linked collagen), bovine fluid collagen contains fibrillar collagen (that is, non-cross-linked collagen). The collagen wound fluid matrix is the most advanced wound care matrix and is a fluid (liquid) variant of the collagen scaffold. However, the uneven geometry of the wound tunnels problematizes its management [97].

5. *Acellular Dermal Matrix (ADM*)

Commercially known as Dermacell, ADM was practiced for many years for wound healing, tissue repairing, and reconstruction. The extracellular matrix performs a vital role in wound healing, as it contributes to structural support and promotes signals to accentuate cellular responses. The dermis of the decellularized donor retains bioactive agents and acts as a scaffold for the repopulation of the host cell. ADM is believed to promote wound healing by increasing vascularization and implementing a barrier against bacteria and a moist wound environment, which enhances cell regeneration [98].

6. Human Amniotic Membrane

The human amniotic membrane was used as a wound covering for more than 100 years. It comes from the deepest layer of the placenta and is formed by epithelial cells, the basal membrane, and the vascular stroma. It produces biologically activated cells and powerful regenerative molecules together with structural support for the extracellular matrix (ECM). Type IV, V, and VII collagens act as a substrate to maintain structural integrity and also promote cell infiltration and wound healing [99]. Table 7 depicts the different cell treatments for DFUs.

##### Honey

Honey is employed since ancient times in treatments for various chronic skin conditions. Honey has antibiotic, antioxidant, and anti-inflammatory features that reduce wounds and burns, granting healing without any adverse outcomes. In modern years, treatment with honey produced great interest as an alternative treatment for DFUs, and different studies evaluated several properties of honey to treat DFUs at different stages [100]. Some animal model studies revealed that honey can stimulate healing. Despite the heterogeneity of the studies and the lack of high-quality evidence, it was inferred that honey dressings are safe, but the data were not adequate to determine its true effectiveness. A current publication compared honey dressings with dressings made of regular saline solution and discovered that honey dressings were more efficient in terms of their healing time and the number of wounds healed at 120 days [101]. However, there was a possible risk of botulism with honey from food products [102].

##### Plant Extracts

Table 8 summarizes the use of plant extracts for the treatment of DFUs. It is well established that traditional Chinese herbal medicine (CHM) is widely practiced and is a substitute for traditional medicine under several conditions. Radix Astragali (R. Astragali) and Radix Rehmannia (R. Rehmannia), are described as promoters of the proliferation of fibroblasts, the central step in wound healing. R. Astragali strengthens the functioning of the “Qi”, which refers to wound healing and muscle regeneration, while R. Rehmannia consists of reducing the heat in the blood, nourishing the “Yin” and enhancing the production of body fluids [103].

In modern years, the importance of chronic skin wound treatments increased, since different plants have different anti-inflammatory and antimicrobial properties that trigger diverse growth factors, cytokines, and chemokines that help in the regeneration of the skin without any problem or adverse effects [104]. For instance, several investigations reported the benefits of plants, such as *Aloe vera*, *Salvia miltiorrhiza*, *Mimosa tenuiflora*, *Alchemilla vulgaris*, *Angelica sinensis*, *Origanum vulgare* L., and *Lavanduela stoechas* L., which are used in a wide range of cosmetic products, such as ointments, creams, and gels, and for alternative uses like the treatment of chronic skin wounds and the regeneration of the skin [105]. *Zicao* is a traditional herbal medicine for wound healing that was used for several hundred years in China. A published survey revealed that arnebin-1, a naphthoquinone derivative, plays the most significant role in the wound healing properties of this plant [106]. In a different study, the effects of arnebin-1 with and without VEGF on the proliferation, migration, and formation of HUVEC tubes was investigated in vitro, as well as the effect of its topical application (in the form of an ointment) on wound healing in an alloxan model of a cutaneous puncture wound [107]. Arnebin-1, in conjunction with VEGF, exerted a synergistic pro-angiogenic effect on HUVECs and accelerated the healing process of diabetic wounds [107].

Oral doses of extracts of citrus peel (lemon, grapefruit, and orange) in diabetic rats were evaluated to examine if these extracts could be beneficial in the regeneration of ulcers. The authors obtained positive outcomes in the reduction of glucose in the blood and wound healing rate and introduced this therapy as a potential therapeutic alternative for the treatment of DFUs [108]. Pawar et al. (2006) reported the application of a methanolic extract of *Sida cordifolia* Linn., an Indian native plant, in a hydrogel formulation, where they assessed its healing features in the wounds of diabetic rats and found very interesting benefits in the cicatrization of diabetic wounds [109]. A clinical case analyzing a combination of two plant extracts, *Hypericum* (*Hypericum perforatum*) and neem oil (*Azadirachta indica*), in the treatment of advanced DFUs was reported by Labichella et al., who noted that the fusion of these extracts helped to decrease the dimension of the ulcer, thereby increasing the granulated tissue and remodeling the skin tissue in the lesion [110]. A year later, the same group of researchers conducted another study using the same combined extracts (Hyperoil) to evaluate the improvement of the glycemic control and peripheral microvascular circulation in neuropathic patients with advanced DFU with positive results [111].

*Momordica charantia* fruit in an ointment base was evaluated for the healing acceleration of diabetic wounds in diabetic male (Sprague-Dawley) rats. The results showed antidiabetic and healing qualities with an increase in the transforming expression of TGF-β [112]. Kiwi fruits also showed antimicrobial and pro-angiogenic features on neuropathic DFUs [113]. Finally, the impact of topical olive oil on the healing of DFUs showed a significant decrease in the DFU area [118], whereas a combination of the neem and Haridra plants to treat wounds that do not normally heal conferred healing effects when combining neem in its topical form with Haridra via oral presentation [120].

#### 3.5.3. Ozone Therapy

From the complexity of DFUs and the lack of oxygenation in the injuries came the idea of using ozone as a therapy, which is administered in diverse formulations as ozonized oils (for example, sunflower or olive oil) [122] or in a mixture of oxygen and ozone, administered directly to the wound. Ozone has antimicrobial activities when applied directly to chronic wounds, and the decomposition of oxygen in the lesions allows the activation of distinct endogenous growth factors, supporting wound healing [123]. However, this treatment has limitations because it can cause unfavorable effects if its application is excessive [124]. A past study described a clinical case in which intracellular ozone injection was practiced on a patient with DFUs in an advanced stage of gangrene. The ozone injection therapy caused unfavorable effects; thus, the patient had to change therapy. Consequently, the authors could not confirm if the ozone therapy was beneficial for the treatment of DFUs. Another clinical case demonstrated the misuse of ozone therapy for the treatment of advanced DFUs and, thus, highlighted the development of better studies and training to implement this therapy in patients with DFU [125]. On the other hand, the efficacy of ozone therapy in patients with superficial DFUs was evaluated in 47 subjects. The authors observed a positive clinical outcome in the mitigation of the wounds, reducing the time of treatment and hospital stay, thereby supporting the use of ozone therapy in the treatment of chronic wounds caused by diabetes [126]. Another study reported a combination of oxygen and ozone as a therapy for DFUs in stages 2–4, according to the Wagner classifications, which evaluates the presence of various growth factors that regulate the decrease in the wound’s area and heal ulcers. VEGF, TGF-β, and PDGF were expressed. The combination of oxygen and ozone was more effective (92%) than the control group (64%). These studies showed that ozone therapy can be used to treat DFUs, but more investigations are required to precisely define when this therapy can be practiced and its risks and benefits [127].

#### 3.5.4. Devices

A device is an instrument, apparatus, implant, or other similar article that (1) is intended to be employed in the diagnosis, cure, mitigation, treatment, or prevention of diseases, and (2) aims to affect the structure or function of the body but does not achieve its primary purposes through chemical actions within the body and does not depend on being metabolized to achieve its intended purpose.

The principal distinctive characteristic between a medicine and a device is whether it works chemically and requires metabolism to create its results. A recent study developed a system made of a latex template and a red light-emitting diode (LED) device to provide mechanical support for a DFU healing latex sheet. Their outcomes suggested that this proposed method may be promising as a future therapy to treat DFUs but emphasized that more studies are required to efficiently apply their method [128].

There is a special shoe device used to treat and cure DFUs. In a previous study, seven patients with a metatarsal head ulcer were evaluated. The therapeutic shoe was employed day and night, and a plastic seal was used as a sign that the shoe was not removed. The effects achieved were beneficial. All the ulcers healed in a median of 56 days, and only one patient exhibited complications due to this therapy. More examination is needed to judge the effectiveness and safety of non-extractable devices to be used in the treatment of DFUs [129]. Another author examined the use of extracorporeal shock waves (ESWTs) for the treatment of DFUs. They found that the study was successful in using ESWTs without any unwanted side effects. Nevertheless, more research and more representative randomized controlled clinical trials are required to establish the effectiveness and safety of ESWT therapy [130].

Another reference using ESWTs for the treatment of DFU infections in a randomized controlled clinical study examined 38 patients with 45 DFUs divided into two groups (the ESWT and the control group) for 20 weeks. The authors showed that the group receiving treatment with ESWTs experienced a decrease of the wound and healing of the ulcers without any adverse effect from the application of this therapy [131]. A beneficial effect of ESWTs on DFUs was also reported as a consequence of the reduction of ulcers (34.5%) over a period of three weeks. However, the authors did not report a complete healing of the DFUs, suggesting the realization of a new randomized clinical study that does not present as many vulnerabilities as those in their study [132]. On the other hand, another author reported that hyperbaric oxygen therapy (HBOT) was applied to improve or heal chronic wounds in patients with diabetes, with only seven studies that involved their inclusion criteria. However, the authors did not find any report that would support the use of HBOT for chronic wounds in patients with diabetes (specifically DFUs). Some authors reported the use of HBOT for the infection and scarring of DFUs and others reported a reduction in amputations. Consequently, more research is required to confirm the use of this therapy in the chronic wounds of diabetic patients [133].

The use of HBOT for the treatment of DFUs in a comparative study was evaluated between a group that received HBOT and another group that only received standard therapy. Their results showed a significant incidence of DFU healing in patients who were treated with HBOT of approximately 73.3%, compared to patients who received standard therapy, which was only 13.3% over eight weeks. These results, therefore, recommend the use of this therapy for the treatment of DFUs [134]. Investigations of the cost, benefit, efficacy, and safety were carried out for HBOT. However, it has not been possible to conclude HBOT’s mechanism of action for the treatment of chronic wounds in diabetic patients, specifically for DFUs. There is no assessment of whether its cost is lower than the standard therapy already used for the treatment of DFUs, as well as doubts regarding its safety and efficacy for the treatment of DFUs. Therefore, more research is required to address these points and provide more information on the use of this therapy [135].

#### 3.5.5. Nanomedicine

Recently, nanomedicine became a very interesting alternative for the treatment of many conditions, including DFUs. There are two main categories of nanomaterials used in wound healing: those that exhibit intrinsic positive properties for wound treatment and those employed as delivery vehicles for therapeutic agents [136]. Two of the most common nanomaterials used for their intrinsic antibacterial activity are silver and cooper, which were tested in a variety of animal and human models. Since silver is extensively used to treat bacterial infection and to prevent wound sepsis due to its well-known antimicrobial properties, a scientific group in Mexico reported the application of silver nanoparticles (AgNPs) (Argotiv) to the wound area over a week and observed a reduction in the diameter and depth of the wound [137]. Another study reported the use of a nanomembranous triple-layered wound patch for the potential treatment of DFUs [138]. This patch consisted of polyacrylic acid (PAA) as the skin-contacting layer, polyvinyl pyrrolidone (PVP) as the middle layer, and polycaprolactone (PCL) as the outermost layer. The PVP layer was loaded in situ with an antibiotic (ciprofloxacin, CFX) and a release of 30% to 60% of CFX was observed in all patches within the first 6 h, followed by a constant (linear) release during the first 48 h. This represented a controlled and one-step treatment of DFUs [138]. Another study offered a list of several polymers used to transport active principles to the wound region; these polymers had a certain degree of success and positive results against DFUs [139]. Gelatin microspheres carrying FGFb in a mice model demonstrated a significant decrease in the rate of infection and accelerated fibroblast proliferation [140]. Likewise, nanofibers carrying curcumin in a diabetic mice model were able to improve the rate of wound closure [141]. DNA particles were used to activate some pathways and ameliorate the outcome of a condition. For example, in a study in mice, the authors reported that the use of DNA nanoparticles (that acted as an antagonist for the stimulator of the interferon gene pathway) could delay the onset of type 1 diabetes and decrease its incidence when they were applied before the onset [142]. In another diabetes complication, diabetic nephropathy, the activity of crocetin-loaded PLGA nanoparticles was analyzed. The results revealed an increase in the deposition of crocetin content in different tissues (principally the kidney and liver) and plasma against the control group [143]. This field has the potential to grow, but there must be further studies to define a gold-standard treatment that implements these kinds of nanomaterials.

#### 3.5.6. Others

##### Energy-Based Therapies

Energy-based therapies utilize technology to externally stimulate the healing of wounds. The modalities that are currently studied include electrical stimulation, shock wave therapy, electromagnetic therapy, laser therapy, and phototherapy [144].

##### Larval Therapy to Treat Ulcers

Larval therapy is practiced on chronic wounds for the removal of necrotic tissue and can stimulate the development of granulated tissue and kill bacteria that cause infection. This therapy is also useful for DFUs that presents a problem due to antimicrobial resistance. Studies were reported with the use of *Chrysomya megacephala*. Recently, these larvae were used for 43 days, resulting in a favorable reduction in the damaged tissue and decreasing the area of the ulcer in a 74-year-old patient [145]. Another study showed the use of the larva *Lucilia sericata*, finding that this type of larva produced secretions with an antimicrobial action in chronic wounds with infections that could not be healed. The larva *Lucilia sericata* has antimicrobial peptides with a broad spectrum against infections and can be considered a new anti-infective therapy [146]. In spite of the FDA’s approval of therapy with larvae, based on different studies that found it to heal wounds by increasing endothelial proliferation and triggering angiogenesis, in conjunction with its antimicrobial action, more research is needed to establish the ultimate role of larval therapy in wound healing [147].

## 4. Collateral Effects of Antimicrobials in Different DFU Therapies

In certain types of DFUs, a patient may exhibit an infection originating from microorganisms that enhances the difficulty of the treatment. In current years, a greater resistance of microorganisms to these many antibiotic therapies was noted. The rise of varied options for the treatment of DFUs (cells, growth factors, or natural compounds) should also include the degree of infection to achieve a short-term increase in tissue regeneration and total wound healing, which is not affected by any infection [148]. Because of these aforementioned issues, it is unknown whether treatment with antimicrobials affects the effectiveness of the use of these new therapies in the healing of DFUs [21]. So far, it is not confirmed that the application of various antimicrobials influences the mechanism of action of different biological therapies or the use of different devices. Instead, different models were generated for their collective use as collagen or sulfate beads for the local discharge of antimicrobials, which obtained positive results in the treatment of infection and scarring [149,150]. However, further investigations are required to guarantee that the activity of antimicrobials improves efficiency, in combination with other therapies, against the diverse microorganism populations found in DFU infections. Table 9 displays the current treatments for DFUs (drugs, devices, and biologics) that are FDA-approved [151,152].

## 5. Conclusions

Several therapies are being studied (biological, devices, drugs, etc.), which, over the years, became very important in the treatment of chronic wounds caused by diabetes, particularly for the treatment of DFUs, which are a global health issue and pose a big burden on a patient’s quality of life. Traditional DFU treatments should not be ceased, and their management according to guidelines needs to be retained. The different studies mentioned in this paper determined the relevant action of the different therapies, such as the use of PDGF, which showed an increase in the rate of wound healing. Some wave shock devices also helped wound healing, as well as stem-cell therapy or the use of natural products, such as honey, which has biological antimicrobial activity. The use of plant extracts, such as *Aloe vera*, shows great antimicrobial promise in the healing of DFUs as well as nanoparticles that have an intrinsic antibacterial activity (like AgNPs) or those that act as delivery vehicles to carry some antibiotics to the wound section (like nanofibers and nanomembranes). However, there is no support for some of these therapies by the FDA because they do not meet the principal purpose for complete closure of the wound; thus, more structured studies are required to confirm the effectiveness of these therapies. Further investigations are required to establish the real benefits of the combined use of cells, genes, devices, nanomaterials and plant extracts as therapies against the different stages of DFUs. These results may help to standardize the treatment of DFUs in the most effective way possible, thereby granting a better quality of life to the patient and their family.

## Figures and Tables

**Table 1:** Classifications of diabetic feet. Comparison of clinical variables according to the criteria recommended by the American Diabetes Association (in terms of management standards, etiology, size, and depth, as well as damage to the deep neighboring structures, degree of infection, evaluation of the vascular and nervous system, and systemic involvement) [13].

Classification		MEGGIT-WAGNER	ANM-SEGAL	TEXAS	S (AD) SAD ^1^	SSS ^2^	GIBBONS	PEDIS	SEWSS ^3^	WIFI ^4^
Etiology	Vascular	-	√	√	√	?	-	√	√	√
	Neurological	-	√	-	?	?	-	√	√	-
	Neuroischemic	-	√	?	-	?	-	-	-	-
Size		-	√	-	√	-	-	√	√	√
Depth		√	√	√	√	-	√	√	√	√
Changes in bone structure		-	√	√	-	-	-	√	√	-
Infection	Cellulite	-	√	?	√	√	√	√	√	√
	Abscess	-	√	?	-	√	√	√	√	√
	Osteomyelitis	√	√	?	√	?	√	√	√	√
	Degrees of severity	-	√	-	?	-	-	√	√	√
Topography		?	?	?	?	?	?	-	√	?
Edema		-	-	-	-	-	-	-	√	-
Healing phases		-	-	-	-	-	-	-	√	-
Metabolic state		-	√	-	-	-	√	-	√	√

^1^ Size (area/depth), sepsis, arteriopathy, denervation. ^2^ Simple staging system. ^3^ Saint Elian wound scoring system. ^4^ Wound ischemia foot infection. √ = Included; - = not included; ? = difficult or doubtful in the identification of criteria of these classifications. Classification systems include Wagner, PEDIS (perfusion, extent, depth, infection, and sensation), University of Texas, WIFI (wound, ischemia, and foot infection by The Society for Vascular Surgery Lower Extremity Limb Classification System), IDSA (Infection Diseases Society of America), and the Saint Elian Wound Score System (SEWSS), which combines scores for various elements of diabetic foot characteristics, such as anatomy, ischemia, infection, neuropathy, edema, and tissue affection.

**Table 2:** Microbiota in diabetic foot ulcers (DFUs). MSSA—methicillin-susceptible *Staphylococcus aureus*; MRSA—methicillin-resistant *Staphylococcus aureus*.

Feature	Gram-Positive Bacteria	Gram-Negative Bacteria	Anaerobes	Reference
Main bacteria found in DFUs	1. *Staphylococcus aureus (MSSA and MRSA)* 2. *Streptococcus* β-hemolytic	1. *Pseudomonas aeruginosa* 2. *Streptococcus* β-hemolytic 3. *Proteus* spp.	1. *Peptostreptococcus* spp. 2. *Bacteroides* spp. 3. *Prevotella* spp. 4. *Clostridium* spp.	[23,24,27,28,29]
Location of wound	Superficial wounds	Superficial wounds	Deep wounds	[24,30,31]
Geographical location	Occidental countries	Eastern and warmer countries	Global	[24,29,32]
Diabetic population	Non-predominance	Predominance	Present	[33]
Non-diabetic population	Predominance	Non-predominance	Present	[33]

**Table 3:** Bacteria isolated from diabetic foot infections (DFIs) and their susceptibility to antibiotics by geographic region.

Bacteria Isolated from DFI	Less Efficient Antibiotic	More Efficient Antibiotic	Geographic Region	Reference
Total isolate	Cephalosporin (ceftazidime, ceftriaxone, cefuroxime), carbapenem (aztreonam)	Not studied	Bangladesh	[32]
Gram-positive	Penicillin, dicloxacillin, and vancomycin	Levofloxacin, cefalotin	Mexico	[34]
Gram-negative	Cefalotin, penicillin, and vancomycin	Amikacin	Mexico	[34]
Anaerobes	Clindamycin, penicillin, and cefoxitin	Imipenem and metronidazole	India	[31]
Gram-negative	Not studied	Piperacillin/tazobactam	India	[31]
Gram-positive and Gram-negative	Not studied	Imipenem	Brazil	[23]
Gram-negative	Not studied	Gentamicin	Brazil	[23]

**Table 4:** Biological products that work in wound repairing for the treatment of DFUs: growth factors and other non-growth factor proteins.

Biological Product	Administration	Reference
Growth factor derived from platelet-BB	Local	[53,54]
Fibroblast growth factor β	Intralesional	[55]
Epidermal growth factor	Intralesional	[56]
Vascular endothelial growth factor	Intramuscular	[53]
Granulocyte colony-stimulating factor	Systemic	[56]
Recombinant human epidermal growth factor	Intralesional	[43]
Insulin	Local	[59]
Neuropeptides	Local, Systemic	[60]
C-reactive protein	Systemic	[61,62,63]
Procalcitonin	Systemic	[61,62,63]
Neurotensin	Systemic	[61,62,63]

**Table 5:** Properties that characterize antibiotics and antimicrobial peptides [68].

Characteristic	Conventional Antibiotics	Antimicrobial Peptides
Spectrum of activity	Bacteria (selectivity)	Bacteria, fungi, viruses, tumors
Objective	Class specific (plasminogen-binding peptide “PBP”, topoisomerase, ribosomes)	Relatively non-specific, multiple objectives
Resistance	After few passes with minimum inhibitory concentration)	Generally, cannot be selected directly; multiple passes are required for minimum inhibitory concentration; specific proteases
Related activities	Few	Include anti-endotoxic mechanisms and increase inn immune response
Pharmacokinetics	It varies	Short average life by proteolytic degradation
Toxicology	Tends to be safe	No toxicities of topical use are known
Production cost	It varies	Expensive, via processes of chemical synthesis

**Table 6:** Classification of the antimicrobial peptides of mammals [68].

Structure	Peptide	Organism	Activity
Linear helical	Cecropin P	*Sus scrofa*	Antibacterial
	Seminalplasmin	*Bos Taurus*	Antibacterial
Non-helical linear	Bac5	*Bos taurus*	Antibacterial
	Indolicidin	*Bos Taurus*	Antibacterial
Cyclic with one disulfide	Bactenecina	*Bos Taurus*	Antibacterial
Cyclic with two or more disulfides	B-defenders 1, 2, 4	*Bos taurus.*	Antibacterial
	Cryptidine 1, 2, 4, 5	*Mus musculus*	Antibacterial
	Defenders *NP*-1, 2, 3A, 3B	*Oryctolagus cuniculus*	Antibacterial/antifungal
	Defenders *HNP*-1, 2, 3, 5, 6	*Homo sapiens*	Antibacterial/antifungal
	Defenders *MCP*-1	*Oryctolagus cuniculus*	Antibacterial
	Protegrin I, II, and III	*Sus scrofa*	Antibacterial/antifungal
	*TAP*	*Bos Taurus*	Antibacterial/antifungal

**Table 7:** The action of some biological products used in wound repairing for the treatment of DFUs: bioengineering and cell culture.

Biological Product	Action	Reference
Grafting (bioengineering)	Promotes wound healing through the addition of extracellular matrices that induce growth factors and cytokines	[96]
Culture of fibroblasts	Creates a three-dimensional dermis that is replaced as a graft; it is used to treat non-ischemic ulcers	[95]
Culture of fibroblasts/keratinocytes	Creates a three-dimensional dermis that replaced as a graft; it is used to treat non-ischemic and ischemic ulcers	[96]
Bovine fluid collagen	It is a well-refined fluid fibrillar bovine collagen; unlike normal collagen in biological scaffolds (cross-linked collagen), it contains fibrillar collagen, that is, non-cross-linked collagen	[97]
Cell dermal matrix	It was used for several years for wound healing, tissue repair, and reconstruction	[98]
Human amniotic membrane	It is used as wound coverage	[99]

**Table 8:** Plant extracts used in wound repairing for the treatment of DFU infections.

Plant Extract	Presentation	Route of Administration	Action	Reference
Arnebin-1	Unguent	Local	Antidiabetic and healing properties	[107]
*Momordica charantia*	Unguent	Local	Antidiabetic and healing properties	[112]
Kiwi	Slices of kiwi	Local	Antimicrobial and pro-angiogenic properties	[113]
*Aloe vera*	Gel	Local	Antimicrobial and pro-angiogenic properties	[114,115,116]
Extracts of citrus peel (lemon, grapefruit, and orange)	Liquid formula	Oral	Antimicrobial and pro-angiogenic properties	[108]
*Sida cordifolia* Linn.	Hydrogel	Local	Antimicrobial and pro-angiogenic properties	[109]
Polyherbal	Cream	Local	Antimicrobial and pro-angiogenic properties	[117]
Olive oil	Topic	Local	Antimicrobial and pro-angiogenic properties	[118]
*Nigella sativa*	Gel	Local	Antimicrobial and pro-angiogenic properties	[119]
Neem and Haridra	Liquid formula, gel	Local, oral	Antimicrobial and pro-angiogenic properties	[120]
Hypericum and neem oil	Unguent	Local	Antimicrobial and pro-angiogenic properties	[110]
*Tragia involucrata*	In vitro study	-	Antimicrobial properties	[111,121]

**Table 9:** DFU therapies that are Food and Drug Administration (FDA)-approved.

Type of Therapy	Pharmaceutical Form	Route of Administration	Advantages	Limitations	Reference
Becaplermin	Gel	Topical	Stimulates different growth factors useful in the treatment of DFUs	Short half-life time	[153]
Cell therapy	Injection or gel	Locally	Stimulates different cellular mechanisms for the regeneration of chronic wounds	Short half-life time	[154]
Collagenase clostridial	Ointment	Topical	Easy application, minimal blood loss, and proliferation of endothelial tissue	Burning, exudation, and inflammation	[155]
Dermapace system	Device	Local shock waves	Stimulates the wound mechanically, for the removal of damaged tissue	Secondary side effects (pain, bruises, etc.)	[156]
Deferoxamine	Injectable	Locally	Reduction of ulcers area in less time	Adverse reactions and its lifetime is short	[157]
Granulox	Spray	Topical	Accelerating the healing of chronic wounds	Short half-life time	[158]
Omnigraft	Device	Topical	Potential for improvement in the DFU	New infections, swelling, and new ulcers, or existing ulcers that would worsen	[156]
Piperacillin/tazobactam (Zosyn, Pfizer)	Injectable	Locally	Wide spectrum advantage in infections and low nephrotoxicity	Adverse reactions may include diarrhea	[159]
Provant	Device	Locally	It is useful in pressure ulcers	There is little evidence of its efficacy	[160]

## Supplementary Materials

The following are available online at https://www.mdpi.com/2079-6382/8/4/193/s1: Table S1: Conventional antibiotic therapies according to the DFU infection degree.
