# Supplementary material for: Diabetic Foot Ulcers: Current Advances in Antimicrobial Therapies and Emerging Treatments
Source: Antibiotics (Basel). 2019 Oct 24;8(4):193. doi: 10.3390/antibiotics8040193 (PMC6963879; doi:10.3390/antibiotics8040193)
Supplement: Supplementary file 1 [file antibiotics-08-00193-s001.pdf]

antibiotics

MDPI

-Additional material-

Review

Diabetic foot ulcers: current advances in antimicrobial therapies and emerging treatments

Jesus M Ramirez-Acuña[1†], Sergio Cardenas[1†], Pedro Marquez-Salas[1, 2], Idalia Garza-Veloz[1, 2*], Aurelio Perez-Favila[1,2], Miguel Cid-Baez[1], Virginia Flores-Morales[3], and Margarita L Martinez-Fierro[1, 2*]

[1] Molecular Medicine Laboratory. Unidad Academica de Medicina Humana y Ciencias de la Salud, Universidad Autonoma de Zacatecas. Carretera Zacatecas-Guadalajara Km.6. Ejido la Escondida, C.P. 98160 Zacatecas, Mexico
[2] Unidad Academica de Ingenieria Electrica, Universidad Autonoma de Zacatecas, Jardín Juárez 147, Centro, 98000 Zacatecas, Mexico
[3] Laboratorio de Sintesis Asimetrica y Bioenergetica (LSAyB). PA de Ingeniería Química, UACQ, Universidad Autonoma de Zacatecas. Carretera Zacatecas-Guadalajara Km.6. Ejido la Escondida, C.P. 98160 Zacatecas, Mexico.
† Equal contributors
* Correspondence: idaliagv@uaz.edu.mx (I.G-V); margaritamf@uaz.edu.mx (M.L.M-F)

Additional Table S1. Conventional antibiotic therapies according to the DFU infection degree.

Infection severity (IDSA)	Recommended antibiotic/ therapy regimen	Bacteria coverture	Monitoring	Reference
Uninfected	N/A	N/A	N/A	N/A
Mild	Topical antiseptics + oral antibiotics<br>- Povidone iodine 10% solution<br>- Chlorhexidine<br>- Acetic acid 5%<br>- Silver compounds	Broad-spectrum activity, antifungal and antiviral properties.	Thyroid dysfunction (povidone iodine 10%)<br>Ear toxicity (chlorhexidine)	[1]
Moderate	Topical antiseptics + oral antibiotics<br>- B-lactams (Amoxicillin-clavulanate, Ampicillin)<br>- Dicloxacilline<br>- Cephalosporin's (Cephalexin, Cefoxitin, Ceftriaxone, Ceftazidime).<br>- Quinolones (Ciprofloxacin, Levofloxacin).<br>- Metronidazole<br>- Clindamycin	Activity against Gram +, Gram -, *S. aureus* MSSA, anaerobes.	Allergic reactions<br>QT prolonging (Quinolones, clindamycin)<br>Nephrotoxicity (Vancomycin)	[2–4]
Severe	Combination of Intravenous	Broad	Allergic	[2–7]

antibiotics

MDPI

	antibiotics<br>- Piperacillin/tazobactam + Clindamycin<br>- Carbapenems (meropenem, imipenem, ertapenem)<br>- Ampicillin/Sulbactam<br>MRSA<br>- Vancomycin<br>- Linezolid<br>- Daptomycin<br>*P. aeruginosa*<br>- Cefepime<br>- Ceftazidime<br>- Meropenem<br>- Piperacillin/tazobactam<br>Anaerobes<br>- Metronidazole<br>- Clindamycin<br>Osteomyelitis<br>- Quinolones<br>- Linezolid	spectrum (Gram +, Gram -), MRSA, *P. aeruginosa*, anaerobes.	reactions<br>QT prolonging (Quinolones, clindamycin)<br>Nephrotoxicity (Vancomycin)	
Term of treatment (IDSA)	*Mild infections*: 1–2 weeks' treatment.<br>*Moderate-Severe infections:* 2–3 weeks' treatment.			[8]
Assessment of wound treatment	Every 2 weeks and at every dressing change.			[2]

References

1. Alavi, A., et al., *Diabetic foot ulcers: Part II. Management.* Journal of the American Academy of Dermatology, 2014. **70**(1): p. 21.e1-21.e24.
2. Tayeb, K.A., et al., *Managing infection: a holistic approach.* J Wound Care, 2015. **24**(Sup5b): p. 20-30.
3. Smith, K., et al., *One step closer to understanding the role of bacteria in diabetic foot ulcers: characterising the microbiome of ulcers.* BMC Microbiology, 2016. **16**: p. 54.
4. Aumiller, W.D. and H.A. Dollahite, *Pathogenesis and management of diabetic foot ulcers.* Journal of the American Academy of PAs, 2015. **28**(5): p. 28-34.
5. Saseedharan, S., et al., *Epidemiology of diabetic foot infections in a reference tertiary hospital in India.* Brazilian Journal of Microbiology, 2018. **49**(2): p. 401-406.
6. Roberts, A.D. and G.L. Simon, *Diabetic foot infections: the role of microbiology and antibiotic treatment.* Semin Vasc Surg, 2012. **25**(2): p. 75-81.
7. Karri, V.V.S.R., et al., *Current and emerging therapies in the management of diabetic foot ulcers.* Current Medical Research and Opinion, 2016. **32**(3): p. 519-542.
8. Everett, E. and N. Mathioudakis, *Update on management of diabetic foot ulcers.* Annals of the New York Academy of Sciences, 2018. **1411**(1): p. 153-165.
